# Teacher-Rated School Leadership and Adolescent Gambling: A Study of Upper Secondary Schools in Stockholm, Sweden

**DOI:** 10.3390/ijerph18189660

**Published:** 2021-09-14

**Authors:** Gabriella Olsson, Bitte Modin, Sara Brolin Låftman

**Affiliations:** Centre for Health Equity Studies (CHESS), Department of Public Health Sciences, Stockholm University, SE-10691 Stockholm, Sweden; bitte.modin@su.se (B.M.); sara.brolin.laftman@su.se (S.B.L.)

**Keywords:** contextual, gambling, multilevel risk gambling, school, youth

## Abstract

So-called “effective schools” are characterised by properties such as a strong and purposeful school leadership and a favourable school ethos. In a previous study we showed that a school’s degree of teacher-rated ethos was inversely associated with student gambling and risk gambling. Building on these findings, the current study aims to examine the associations that teachers’ ratings of the school leadership share with gambling and risk gambling among students in the second grade of upper secondary school in Stockholm (age 17–18 years). Data were drawn from the Stockholm School Survey and the Stockholm Teacher Survey with information from 5191 students and 1061 teachers in 46 upper secondary schools. School-level information from administrative registers was also linked to the data. The statistical method was two-level binary logistic regression analysis. Teachers’ average ratings of the school leadership were inversely associated with both gambling (OR 0.96, 95% CI 0.93–0.998, *p* = 0.039) and risk gambling (OR 0.94, 95% CI 0.89–0.99, *p* = 0.031) among upper secondary students, whilst adjusting for sociodemographic characteristics at the student and the school level. The findings lend further support to the hypothesis that characteristics of effective schools may reduce students’ inclination to engage in gambling and risk gambling behaviours.

## 1. Introduction

Adolescent problem gambling has been declared as an important public health issue [1]. Having gambling problems in adolescence can lead to many negative social, educational, economic and health-related consequences [2]. Gambling problems in youth may also increase the risk of re-occurring gambling problems later in life [2]. Acting early to prevent youth from getting gambling problems is thus important [2,3]. In Sweden, gambling is included in the Social Services Act and the Social Welfare Board has been tasked to actively counteract the abuse of gambling for money among children and young people [4]. However, and despite being illegal, 25 percent of Swedish 17-year-olds have gambled for money. For about three percent of them the activity can be classified as at risk or problem gambling [4]. Accordingly, enhanced knowledge about the determinants of adolescent gambling has been called for [2]. While earlier research on adolescent gambling has largely focused determinants at the individual- and the family-level studies, expanding this knowledge by exploring the influence of other contexts on youth gambling areparticularly needed [3,5]. Both theory [6] and research into other risk behaviours [7,8,9] suggest that such an approach may be highly relevant. In the current study, we will draw upon elements from the field of effective school research to investigate the links between conditions in school and adolescents’ gambling.

As suggested by Bronfenbrenner [6], a child’s development is the result of the influence of multiple social contexts acting at different societal levels. The school is one such an important context. School provides opportunities for children and adolescents to create social relationships outside the family, essentially with peers, but also with teachers and other adults who may provide social support and act as role models. Hence, although the main role of school is to transmit knowledge and academic skills it also serves a significant role in shaping young peoples’ values and behaviours.

The Swedish school system has in the last decades gone from being a unified school system with state run schools to becoming one of the world’s most decentralized systems where the student has a great opportunity to choose a school and where a larger proportion of the schools are independent schools [10]. Together with a parallel process of increased school segregation along ethnic and socioeconomic lines these structural changes have greatly affected the conditions under which schools operate. It has been suggested that this development not only has resulted in a more segregated school system, but that it also has affected schools’ capacity to provide equal learning environments [11].

Schools’ capacities to form a favourable social arena for the students can be expected to be related to their degree of “school effectiveness” [12]. Research on school effectiveness dates back to the beginning of the 1970s and was then a reaction to work that proclaimed that family background and school composition, rather than characteristics of the school per se, determined a student’s school performance [13]. The new research field highlighted schools’ ability to overcome disadvantages associated with the student intake by improving their contextual features [14]. In line with these ideas, inquiry into so-called “effective schools” has conveyed that certain schools are more successful than others in creating a positive school environment, regardless of the sociodemographic student composition [14,15,16]. The prominent features of effective schools referred to in the literature are many and differ depending on methodological considerations and theoretical starting points [17]. However, a number of features were early identified as common and re-occurring among particularly successful schools [14,15,16]. These features include high expectations of students, a strong educational leadership, an emphasis of basic skills, regular monitoring of students’ achievements, an orderly environment, constructive feedback from teachers, strong parent–school relationships and a positive school ethos. The latter refers to the norms, attitudes and behaviours that characterise the social interaction among teachers and students [14,15,16,18]. In more recent research on effective schools, the crucial role of a strong and purposeful school leadership for creating favourable organisational conditions has been emphasised [11,19]. In such models, the underlying idea is that higher-level properties should provide the necessary conditions for lower-level processes to come into force [20,21]. For instance, a strong school ethos is imposed from higher levels in the school structure through a purposeful school leadership [22]. The capacity of a school’s leadership is thus seen as central for the effectiveness of the school as a whole. In previous research, school effectiveness features have been linked to higher performance among students [14], but also to less engagement in various health risk behaviours [16,23,24]. In addition, prior studies of the same data material that is used in the current study have shown that higher teacher ratings of the school leadership are associated not only with higher academic performance [11] but also with a lower likelihood of negative behaviours such as truancy [25] and bullying perpetration [26] among students.

Research on the influence of conditions in school and gambling is yet limited [5]. There are, however, some studies available that suggest that contextual conditions [27], including certain contextual conditions in school [5,28] may influence youth gambling. For instance, a study by Lee et al. [5] suggests that gambling is weakly and negatively associated with schools’ suspension rate, while a study by Elgar et al. [28] showed an association between relative deprivation among classmates and symptoms of disordered gambling (i.e., felt like but could not stop gambling, relational problems or problems at school/work caused by gambling). Studies exploring properties of school effectiveness and students’ inclination to engage in gambling are even less common. However, such an approach could potentially point to conditions in school that could be targeted to lower the risk for gambling problems, an approach that has been called for [3]. One exception is a study that was based on the same data as the current one, which showed that a school’s degree of teacher-rated ethos was inversely associated with gambling and with risk gambling among their students [29]. Building on these findings, the present study aims to examine the associations that teachers’ ratings of the school leadership shares with gambling and risk gambling among students, whilst adjusting also for sociodemographic characteristics at the student- and the school level. The assumption is that a purposeful school leadership is a prerequisite for a well-functioning school where the teachers have good opportunities to instruct and support their students, which in turn may affect students’ academic motivation and performance as well as their behaviour. More specifically the following research question has been guiding the study:

Is there an association between schools’ teacher rated school leadership and student gambling and risk gambling once the sociodemographic composition of the school has been controlled for?

## 2. Materials and Methods

### 2.1. Data

The data were drawn from two cross-sectional surveys performed in 2016: The Stockholm School Survey (SSS) and the Stockholm Teacher Survey (STS). The SSS is carried out by Stockholm Municipality every two years, among students in Grade 9 of senior-level schools and in Grade 2 of upper secondary schools in all public schools and in a number of independent schools in Stockholm. Students complete the questionnaires in the classroom. The questionnaires cover topics such as substance abuse, criminal behaviour, and gambling, but also subjective health, relations with parents, and the situation at school. Stockholm Municipality has estimated the response rate in 2016 to 78% [30]. The STS has been conducted on two occasions as part of a research project: in 2014, among teachers in all the senior-level schools that participated in the SSS; and in 2016, among senior-level and upper secondary school teachers in all the schools participating in the SSS. The idea behind the STS was to collect information about school characteristics from teachers on e.g., the school leadership, cooperation and consensus among the staff, and school ethos, and to link these data to information collected among students in the SSS. The STS was carried out as a web survey and the response rate among teachers in upper secondary schools was 58% [31]. To create school-level measures, the mean value of teachers’ ratings at each school was calculated and merged to the student-level data from the SSS. To the data, we also linked administrative register information on schools from the Swedish National Agency for Education. The current study was based on a merged data set of the SSS and the STS, covering 46 schools with information from 5191 students attending Grade 11 (i.e., the second year of upper secondary school: age 17–18 years) and 1061 upper secondary teachers for Grades 10–12 in the corresponding schools. Internal non-response rate in the SSS was 7.4%. Further information on the data is provided in the technical report [31].

Since the Stockholm School Survey is performed anonymously (with no information on personal identification) the data are not subject to consideration for ethical approval, according to a decision by the Regional Ethical Review Board of Stockholm (2010/241-1/5). Ethical approval has been obtained for the Stockholm Teacher Survey (2015/1827-1/5).

### 2.2. Measures

Gambling was based on the question “Have you bought lottery tickets or gambled for money at any time during the last 12 months?”, with the specification “(Scratch ticket, game show lottery, casino, poker, betting on football, horses or the like, also on the Internet)”. The response categories were “no” and “yes”. The measure has been used in previous studies [29,32].

Risk gambling was measured by three questions which were asked to students who had marked that they had been gambling in the past 12 months: “How many times during the last 12 months have you”: (a) “Tried to reduce your gambling?”, (b) “felt restless and irritated if you have not been able to gamble, and (c) “lied about how much you’ve gambled?” The response categories were “never”, “1–2 times”, and “3 times or more”. Participants who marked “at least one” on any of the three items were classified as having engaged in risk gambling. The items have been used in prior studies to measure risk gambling [29,32,33].

School leadership was assessed through teachers’ responses to ten items in the STS: (a) “the management has an interest in pedagogical questions”; (b) “the management shows an understanding of my work problems”; (c) “the school leaders have high expectations of me as a teacher”; (d) “when the management makes decisions on important issues they first discuss it with the teaching staff”; (e) “the majority of teachers’ understanding of school goals and policies align with the management’s”; (f) “the management allows room for teachers’ pedagogical freedom”; (g) “I regularly receive feedback from the management about my performance as a teacher”; (h) “the management is a good support for teachers experiencing difficulties with a class”; (i) “the distribution of responsibility between teachers is clear at this school”; and (j) “this school is led in a good way”. The response categories were on a five-point scale ranging between “strongly agree” (5) and “strongly disagree” (1). Values from all ten items were added to an index with the possible range 10–50, with higher values indicating higher teacher ratings of the school leadership. The measure had good psychometric properties (RMSEA = 0.061; CFI = 0.993; TLI = 0.990) and high internal consistency (Cronbach’s alpha = 0.90). The mean value of the index for each school was used as a school-level measure of school leadership. The measure has been used previously [12,25,26].

A number of sociodemographic control variables were included. At the student-level, we included gender, family structure, parents’ university education, and migration background. At the school-level, we included the school proportion of students whose parents had a post-secondary education and the school proportion of students with a foreign background.

### 2.3. Descriptive Statistics

Table 1 displays descriptive statistics of the study sample. With regards to the school-level variables, the mean value of teacher-rated school leadership was 33.9 (range 24.7–44.6), the mean proportion of students whose parents had a post-secondary education was 51.5% (range 7.0–86.3%), and the mean proportion of students who had a foreign background was 41.1% (range 6.0–95.7%). Among the participants in the study sample, 14.9% reported that they had been gambling during the past 12 months, and 3.4% were classified as risk gamblers. The sample contained 46.6% boys and 53.4% girls. Among the participants, around two thirds lived with two parents in the same household. Two thirds reported having at least one parent with a university education, and about one in ten had lived in Sweden for a period shorter than ten years.

### 2.4. Statistical Method

Given the hierarchical nature of the data with students nested in schools, and since the purpose was to examine the association between school leadership at the school level and gambling and risk gambling at the student level, multilevel analysis was applied. Multilevel analysis takes the hierarchical structure of the data into account by allowing the variance in the outcomes to be separated between higher level units [34], in this case, into student-level variation and school-level variation. The approach is thus ideal for the purpose of the study. Two-level binary logistic regression models were performed using the “xtmelogit” command in Stata, version 15.0 [35]. Odds ratios (OR) and 95% confidence intervals (95% CI) are presented. Additionally, the intra class correlation (ICC) is reported, which in multilevel binary logistic regression provides an approximation of the amount of variation that can be attributed to the higher level. The same analytical approach has been applied in previous studies based on the same data [22,25,26,29].

## 3. Results

To examine the associations that school leadership shares with gambling and with risk gambling, respectively, two separate two-level binary logistic regression analyses were performed, adjusting for the full set of student and school level variables. The results, presented in Table 2, show that school leadership was inversely associated with both gambling (OR 0.96, 95% CI 0.93–0.998, *p* = 0.039) and risk gambling (OR 0.94, 95% CI 0.89–0.99, *p* = 0.031), whilst adjusting for sociodemographic characteristics at the student and the school level. With regards to gambling, the proportion of students with parental post-secondary education was associated with gambling at the student-level (OR 0.99, 95% CI 0.98–0.995, *p* = 0.001). The association with the proportion of students with foreign background was however not statistically significant at conventional levels (OR 0.99, 95% CI 0.98–1.00, *p* = 0.057). Girls were less likely than boys to have been gambling during the past 12 months (OR 0.25, 95% CI 0.21–0.30), whereas students not living with two parents in the same household were more likely to have gambled than those living with two parents (OR 1.18, 95% CI 1.00–1.40). No statistically significant differences were, however, seen for parental university education, or migration background. Risk gambling was inversely associated with the school proportion of students with parental post-secondary education (OR 0.99, 95% CI 0.98–0.999, *p* = 0.029), whereas there was no statistically significant association between the school proportion of students with foreign background and risk gambling (OR 0.99, 95% CI 0.98–1.002, *p* = 0.117). Risk gambling was much less common among girls than among boys (OR 0.08, 95% CI 0.05–0.14). Students not living with two parents in the same household were more inclined to engage in risk gambling compared with those living with two parents (OR 1.41, 95% CI 1.02–1.94). Participants who reported having at least one university educated parent had a lower likelihood of risk gambling (OR 0.63, 95% CI 0.46–0.88) whereas there was no statistically significant difference by migration background.

## 4. Discussion

The ambition with the current study was to explore the association between a school’s degree of teacher rated school leadership and student gambling and risk gambling. Using a data material with combined information from teachers, students, and administrative registers, this study showed that teachers’ ratings of the school leadership were inversely associated with self-reported gambling and risk gambling among upper secondary students, whilst adjusting also for sociodemographic characteristics at the student and the school level. The findings corroborate and extend prior research which has shown that features of school effectiveness are associated with fewer health risk behaviours among the students [16,23,24]. The current study also extends the results of a prior publication based on the same data which showed that a school’s level of teacher-rated ethos was inversely associated with student gambling and risk gambling [29], and thus indicates that features of school effectiveness at different levels of the school organisation may be relevant for students’ inclination to engage in health risk behaviours.

Overall, the result of the current study reflect well the more recent research on effective schools in which the crucial role of a strong and purposeful school leadership for creating favourable organisational conditions has been emphasised [19]. Common for this line of research is that the influence of school leadership on student outcomes is generally regarded as largely indirect [19,21,36]. As such, school leadership is believed to act on student outcomes by its influence on more proximal processes at lower levels in the school organisation. With regards to the association between school leadership and gambling, a number of more proximal processes may thus be at work. While the ambition of the current study has not been to explore the mechanisms at work between school leadership and student gambling and risk gambling, some plausible pathways can be derived on the basis of previous research. It is for instance reasonable to assume [21] that a strong school leadership may be reflected in a school’s ethos, i.e., the attitudes, values and behaviours that characterise the social interaction among teachers and students and that are central in order to prevent the emergence of undesirable behaviours [22] including gambling and risk gambling [29]. Teachers’ attitude to their students is a central component of the ethos concept [37]. It may well be that by providing the necessary conditions for a strong school ethos, a strong school leadership enables more supportive student–teacher relations to take form. That both school leadership and school ethos have previously been linked with higher levels of perceived teacher caring among students support such a notion [38]. In gambling research on the other hand, social connectedness and the importance of healthy and meaningful relationships hasveepeatedly been highlighted as central protective factors [2]. In addition, school connectedness, i.e., one’s feeling of being treated fairly, being close to others, and an integral part of the school, has been found to be associated to gambling severity [39]. It, thus, seems plausible to assume that one of the underlying pathways between school leadership and student gambling may run through school ethos and more precisely positive student–teacher relations. Indeed, positive student–teacher relationships have previously been shown to be negatively associated with student substance use [24] and to combat the risk of heavy drinking among students from risk laden family conditions [40].

It is also plausible that the association between school leadership and gambling can be understood from point of view of students’ future orientation. A more pessimistic future orientation has previously been linked to gambling and risk gambling [32]. Students with a pessimistic future orientation have been found to be more likely to engage in gambling and risk gambling as compared to those with a more optimistic future orientation [32]. It has been suggested that individuals who sense a lack of future prospects are more inclined to seek immediate benefits rather than to invest in behaviour associated with delayed gratification [32]. In a previous study based on the same data as the current one, a strong school leadership has been linked to more optimistic beliefs and feelings about the future among students [12]. A strong leadership may thus, by setting the ground for a well-functioning school, promote factors such as students’ academic motivation and school performance [11] as well as positive student-teacher relations [38] that in turn are likely to awake higher aspirations and confidence for the future among its students.

It is also possible that other, non-observed school contextual aspects are confounders or mediators in the association between school leadership and gambling. A strong school leadership may for instance be a prerequisite for effective school policies with regards to risk behaviours. To learn more about the association between school leadership and student gambling, plausible pathways underlying the association between conditions in school and youth gambling and risk gambling should be further explored.

While the main aim of this study was to investigate the association between school leadership and student gambling, it should be noted that associations were also found for student-level characteristics and gambling as well as risk gambling. Without going into depth with these findings it could be noted that they largely reflect previous research [28,39,41]. For instance, boys were found to be more inclined than girls to engage in gambling and, in particular, risk gambling. In the literature [2], cultural influences that make parents encourage boys more than girls to be involved in gambling has been put forward as possible explanation to the association. Moreover, students not living with two parents in the same household were in the current study found to be more inclined than their counterparts to be engaged in both gambling and risk gambling. While the result is in line with that of other studies [41], research that further explores the relationship between family characteristics, family functioning and gambling has been called for [42]. However, there are evidence suggesting that youth who report family problems and perceive their families as unsupportive [42], youth who experience lack of family cohesion [39] as well as youth who report poor parent attachment [2] are at increased risk for developing gambling problems. In addition, the strong empirical support for these types of family related characteristics and other risk behaviours in adolescence [42], might suggest that they are topics worth further focus also in studies on risk gambling. Finally, in line with the result of other studies in which high parental socioeconomic status has been found to be an early protective factor for the development of gambling problems [2,3], the result of the current study indicated that students not having university educated parent(s) were more likely to be engaged in risk gambling than students with university educated parent(s). To gain a deeper understanding of the aetiology of gambling behaviours, a promising avenue for future research may be to explore how conditions in school interact with these or other known individual risk factors to hinder or facilitate youth gambling. Such an approach has been called for [3] and could further help to identify characteristics of settings outside the family that may be targeted to prevent youth risk gambling, in particular in the face of risk.

Although the data material with combined information from teachers, students, and administrative registers is an obvious strength, there are also limitations. One reservation is that we cannot fully account for the selection of students into schools. Even though the analyses adjust for schools’ sociodemographic characteristics, it cannot be ruled out that there are other, unobserved factors that are associated with both students’ likelihood of attending a certain school and their likelihood of involving in gambling and risk gambling. Whether the school is a public or private school could be one such unobserved factor. In relation to this particular variable, sensitivity analyses however showed no significant effect on student gambling nor did it influence the association between school leadership and gambling. A related concern is the cross-sectional nature of data, which limits our possibilities to draw conclusions about causality. To investigate if characteristics of effective schools make students becoming less inclined to engage in gambling and in risk gambling, longitudinal data are needed. Finally, the fact that our findings were based on data collected among teachers and students in upper secondary schools in Stockholm means that they cannot be generalised to other geographical or national contexts.

## 5. Conclusions

The current study indicated that a strong school leadership may help prevent against students’ inclination to engage in gambling and in risk gambling. In more general terms, such a result provides further evidence to the notion of effective schools by suggesting that properties of effectiveness at different levels of the school organisation have the potential to counteract unwanted behaviours among the students. Further research is needed to gain more in depth knowledge and to discern the processes underlying the association. Exploring mediating mechanisms in school or the interaction between individual background characteristics and school conditions could be possible advantageous avenues for future research. In line with notions of socio-ecological theories [6] our findings further underscore the importance of not only focusing student background characteristics when variation in student outcomes are explored, but also the influence of other contexts. Such an approach could possibly also open up for other and broader school interventions that target conditions in school rather than certain groups of students or certain behaviours. In fact, while most such school conditions have been found to predict multiple educational and health compromising behaviours, it has been suggested [39] that interventions aimed at improving conditions in school may have a greater impact on youths’ long-term development than interventions focusing on only those factors that predict a single negative behavioural outcome. Improving a school’s leadership may thus be one way to promote not only educational motivation but also sound behaviours among youth and positive long-term development, irrespective of the socioeconomic background of the student and the socioeconomic composition of the school.

## Figures and Tables

**Table 1:** Descriptive statistics (*n* = 5191 students in 46 upper secondary schools).

	Mean	s.d.	Min	Max
School level				
School leadership	33.9	3.9	24.7	44.6
Percentage of students with parents with post-secondary-education	51.5	25.1	7.0	86.3
Percentage of students with a foreign background	41.4	21.5	6.0	95.7
Student level	*n*	%		
Gambling	775	14.9		
Risk gambling ^a^	175	3.4		
Gender				
Boy	2421	46.4		
Girl	2770	53.4		
Family structure				
Two parents in the same household	3316	63.9		
Other	1875	36.1		
Parental university education				
No or not known	1730	33.3		
At least one parent	3461	66.7		
Migration background				
≥10 years in Sweden	4713	90.8		
<10 years in Sweden	478	9.2		

^a^*n* = 5139.

**Table 2:** Odds ratios and 95% confidence intervals from two-level binary logistic regression of gambling and risk gambling.

	Gambling		Risk Gambling	
	OR	95% CI	OR	95% CI
School level				
School leadership	0.96 *	0.93–0.99	0.94 *	0.89–0.99
Percentage of students with parents with post-secondary-education	0.99 **	0.98–0.99	0.99 *	0.98–0.99
Percentage of students with a foreign background	0.99	0.98–1.00	0.99	0.98–1.00
Student level				
Gender				
Boy	1.00		1.00	
Girl	0.25 **	0.21–0.30	0.08 **	0.05–0.14
Family structure				
Two parents in the same household	1.00		1.00	
Other	1.18 *	1.00–1.40	1.41 *	1.02–1.94
Parental university education				
No or not known	1.00		1.00	
At least one parent	0.91	0.76–1.08	0.63 **	0.46–0.88
Migration background				
≥10 years in Sweden	1.00		1.00	
<10 years in Sweden	0.84	0.62–1.14	1.35	0.84–2.17
ICC %	4.0		3.8	
*n* (schools)	46		46	
*n* (students)	5191		5139	

** *p* < 0.01 * *p* < 0.05.

## Data Availability

The data are not publicly available. Access to data from the Stockholm School Survey can be applied for at Stockholm Municipality, Sweden. Access to data from the Stockholm Teacher Survey can be applied for at the Department of Public Health Sciences, Stockholm University, Sweden.
